# New Insights into Amino-Functionalization of Magnetic Nanoplatelets with Silanes and Phosphonates

**DOI:** 10.3390/nano12122123

**Published:** 2022-06-20

**Authors:** Jelena Papan Djaniš, Griša Grigorij Prinčič, Andraž Mavrič, Alenka Mertelj, Jernej Iskra, Darja Lisjak

**Affiliations:** 1 Department for the Synthesis of Materials, Jožef Stefan Institute, 1000 Ljubljana, Slovenia; 2 Centre of Excellence for Photoconversion, Vinča Institute of Nuclear Sciences, National Institute of the Republic of Serbia, University of Belgrade, 11351 Belgrade, Serbia; 3 Department for Organic Chemistry, Faculty of Chemistry and Chemical Technology, University of Ljubljana, 1000 Ljubljana, Slovenia; grisa.grigorijprincic@fkkt.uni-lj.si (G.G.P.); jernej.iskra@fkkt.uni-lj.si (J.I.); 4 Materials Research Laboratory, University of Nova Gorica, 5000 Nova Gorica, Slovenia; andraz.mavric@ung.si; 5 Department of Complex Matter, Jožef Stefan Institute, 1000 Ljubljana, Slovenia; alenka.mertelj@ijs.si

**Keywords:** barium hexaferrite, nanoplatelets, coatings, alendronic acid, amino-silane

## Abstract

Magnetic nanoplatelets (NPLs) based on barium hexaferrite (BaFe_12_O_19_) are suitable for many applications because of their uniaxial magneto-crystalline anisotropy. Novel materials, such as ferroic liquids, magneto-optic composites, and contrast agents for medical diagnostics, were developed by specific surface functionalization of the barium hexaferrite NPLs. Our aim was to amino-functionalize the NPLs’ surfaces towards new materials and applications. The amino-functionalization of oxide surfaces is challenging and has not yet been reported for barium hexaferrite NPLs. We selected two amine ligands with two different anchoring groups: an amino-silane and an amino-phosphonate. We studied the effect of the anchoring group, backbone structure, and processing conditions on the formation of the respective surface coatings. The core and coated NPLs were examined with transmission electron microscopy, and their room-temperature magnetic properties were measured. The formation of coatings was followed by electrokinetic measurements, infrared and mass spectroscopies, and thermogravimetric analysis. The most efficient amino-functionalization was enabled by (i) amino-silanization of the NPLs precoated with amorphous silica with (3-aminopropyl)triethoxysilane and (ii) slow addition of amino-phosphonate (i.e., sodium alendronate) to the acidified NPL suspension at 80 °C.

## 1. Introduction

Ferrimagnetic barium hexaferrite (BaFe_12_O_19_) is traditionally used as ceramics in permanent magnet and microwave applications [1]. The existing technologies are mature and only minor advancements are expected. In contrast to this, barium hexaferrite nanoplatelets (NPLs) constitute ferromagnetic liquids in which the nanoplatelets are ferromagnetically coupled [2,3]. These liquid materials are ferromagnetic at room temperature and have opened possibilities for new advanced applications in magneto-optics/photonics, magneto-rheology, bioimaging, water purification, and spin-memory devices [4,5,6]. More applications of barium hexaferrite NPLs have been proposed by tuning their surface chemistry. When coated with a porous phosphonate or biocompatible coating, they can be used for water purification of heavy metals [5] or in medical diagnostics [7] and magneto-mechanical therapy [8], respectively. To ensure such exciting applications, the properties and stability of the functional coatings should be thoroughly studied and understood.

All new applications are based on systems of fully dispersed barium hexaferrite NPLs partly substituted with Sc^3+^ (BSHF), ensuring applicable magnetic properties of very thin (3–5 nm) NPLs with diameters of 20–100 nm [9,10]. BSHF NPLs were dispersed at the highest concentrations in 1-butanol when stabilized by dodecylbenzene sulfonic acid (DBSA) [3,11]. The long-range electrostatic repulsion provided by the DBSa double layer was found to be crucial in suppressing the long-range magnetic dipole attraction between ferrimagnetic BSHF NPLs [12]. BSHF NPLs were also successfully electrostatically stabilized in water by modifying their surface with citric acid or amorphous silica or phosphonate coatings [5,13,14]. The silica coating increased the density of the reactive surface hydroxyl groups, enabling subsequent functionalization by covalent bonding of silanes with specific functionality. Functionalization of metal oxides with alkoxysilanes is very common [15,16]. The alkoxysilanes condense with hydroxyl groups at the oxide surface, ensuring a stable covalent bond [17]. They can bind directly to the surfaces of metal oxides (direct route) or after precoating the oxides with a silica coating (indirect route). The latter ensures a higher surface density of functional groups than the direct route [18]. 

Functionalization of oxide nanoparticles with amine groups enables subsequent functionalization and coupling reactions [15,16,19,20]. However, the amino-functionalization of oxide surfaces is challenging because the amine ligand has to bear an additional anchoring group (e.g., siloxane, phosphonic, or carboxylic) to bond to the oxide surface. Such ligands exist in water up to a relatively high pH in the zwitterion form, at which the amine group deprotonates. In contrast, trialkoxyamino-silanes have only one charge-bearing group (i.e., the amino group), and they cannot form a zwitterion unless they hydrolyze. One of the most used amino-silanes is (3-aminopropyl)triethoxysilane (APTES). When functionalizing oxide nanoparticles with APTES, precise control of the coating conditions (proportion of APTES/nanoparticles, temperature, pH, and solvent) is required to obtain a high density of amino groups on the surface [18,19,21]. Moreover, APTES forms one of the least stable coatings among silanes [22]. Complex structures with several donors and acceptors promote hydrolysis and hydrogen bond formation with surface silanol groups, e.g., the formation of structurally poorly defined inhomogenous multilayer condensation of silane molecules [21,23,24].

Alternatively, bifunctional phosphonates enable simultaneous surface functionalization and strong attachment to the metal oxide surface via the phosphonic group [25,26]. The mode and strength of the acid–surface interaction should be tuned by the pH simultaneously with changes of the acid and surface charges. The possible interaction modes include physical (e.g., electrostatic and hydrogen bond) and chemical (i.e., coordinative) interactions. The coordinative interactions include monodentate, bidentate (mono- and binuclear), and even tridentate modes [25,27]. Since a water molecule is released to bond to the surface of metal ions with the phosphonic group during the condensation, the surface and/or phosphonic groups should be hydroxylated. For example, adsorption of phosphonates and phosphates on goethite was promoted when they were fully/partially protonated, i.e., at acidic pH [28]. Stable phosphonic coatings from various phosphonic acids were also obtained on BSHF NPLs at acidic pH [5]. Coatings of bisphosphonate successfully inhibited the corrosion of biodegradable Zn-based biomaterials [29] and reduced the cytotoxicity of the NaYF_4_-based upconverting nanoparticles, magnetite, etc. [30,31,32]. One such bisphosphonate is alendronic acid (AL) (i.e., (4-amino-1-hydroxy-1-phosphonobutyl)phosphonic acid), an amine-terminated bisphosphonic acid that is supposed to bond strongly with its two phosphonic groups to the surface of metal oxides. Moreover, AL is biocompatible and is used as a sodium salt (Fosamax) for the treatment of osteoporosis [33]. 

Our aim was to study the functionalization of BSHF NPLs with amino groups using APTES and AL and to assess the stability of the coating. We studied the effect of pH and the relative ratio of differently charged species on the coating formation and its stability. The assessment was based primarily on the zeta potential measurement of the coated BSHF NPLs in aqueous suspensions. Namely, the pH values of the isoelectric point of the suspensions should increase with the increasing density of the surface amine groups.

## 2. Materials and Methods

### 2.1. Materials

Barium (II) nitrate (99.95%), scandium (III) nitrate hydrate (99.9%), iron (III) nitrate nonahydrate (98+%), citric acid (99+%), tetraethylorthosilicate (99%), sodium hydroxide (98%), 3-aminopropyl)triethoxysilane (APTES) (98%), and sodium alendronate (a source of AL) (97%) were purchased from Alfa Aesar (Lancashire, UK). Nitric acid was purchased from Sigma Aldrich (Burlington, MA, USA).

Precise concentrations of the metals in the salts were determined with inductively coupled plasma (ICP-OES, Agilent 720, Santa Clara, CA, USA). Deionized water was used in all experiments. Barium hexaferrite nanoplatelets partly substituted with Sc^3+^ (BSHF NPLs) were synthesized hydrothermally as previously described [9], followed by washing with nitric acid and water. The obtained BSHF NPLs were colloidally stable in water after tuning the pH to pH = 2 with nitric acid. 

**Figure 1:**
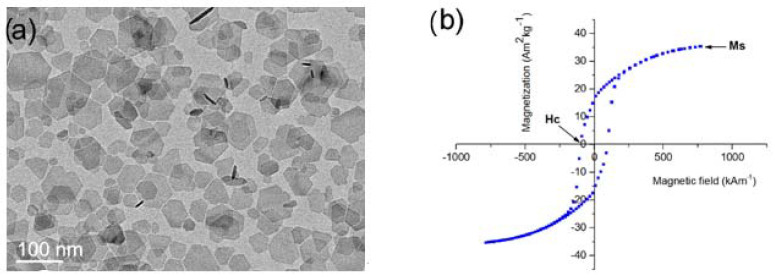
As−synthesized BHSF NPLs: (**a**) TEM image and (**b**) magnetic hysteresis measured at room temperature with saturated magnetization (Ms) and coercivity (Hc).

The BSHF NPLs showed a typical hexagonal plate-like shape with a magnetoplumbite crystal structure (Figure 1a). A selected-area electron diffraction (SAED) of the sample is shown in Figure S1 in the Supplementary Materials. The detailed structural analysis of BSHF NPLs is given in [10]. Figure 1 represents magnetic hysteresis (Figure 1a) with saturation magnetization of Ms~35 Am^2^kg^−1^ and a significant coercivity of Hc = 97 kAm^−1^ typical for hard magnetic materials such as barium hexaferrite (Figure 1b).

### 2.2. Coating BSHF NPLs with APTES

As-prepared BSHF NPLs were first coated with citric acid (CA) and then with silica to produce more stable NPLs. Coatings with silica were prepared with tetraethyl orthosilicate (TEOS) on the surface of the BHF CA using a slightly modified Stöber process. The detailed coating process is described in [14]. NPLs coated only with citric acid are named BSHF-CA, and NPLs coated with citric acid and silica are named BSHF-Si. Coatings with APTES were prepared using 30 mg of dispersed BSHF-CA or BSHF-Si, with dispersion ratios of the volume mixture of water/ethanol/ammonium hydroxide = 1/1/0.06 (total volume 50 mL). The nominal fraction of APTES was set to 5 or 30 molecules/nm^2^ (Table 1). The surface nominal fraction was calculated from the average size of nanoplatelet (approximately 50 nm) and their thickness (4 nm), and detailed calculations are given in the Supplementary Materials. Coatings with APTES are usually prepared at temperatures around 70–90 °C [21], but our reaction mixture aggregated upon heating; therefore, the reaction suspension was stirred overnight at room temperature. The APTES-coated NPLs were sedimented by centrifugation, 2000 rcf for 10 min, and washed five times with a water:ethanol mixture (1:1).

### 2.3. Coating BSHF NPLs with AL

**Scheme 1:**
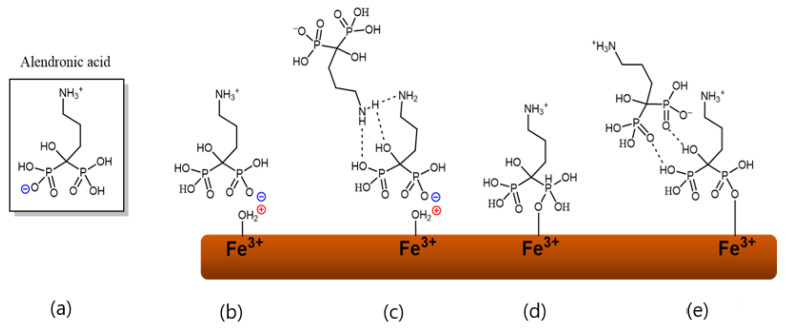
Possible interaction of AL with the BSHF-NPL surface at pH = 2: (**a**) AL structure at pH = 2; (**b**,**c**) physisorption via an electrostatic attraction; (**d**,**e**) chemisorption: Panels (**b**,**c**) show the interaction of a single AL molecule with the BSHF surface. Panels (**c**,**e**) show the interaction of an AL associated with the NPL surface.

The BSHF NPLs thus prepared were coated with AL using the staring aqueous suspension of NPLs (Table 2). Sodium alendronate, a source of AL, was dissolved in water and the pH was adjusted with aqueous solutions of HNO_3_ or NaOH to tune the protonation degree of the AL, in particular, (i) pH 2 to deprotonate only one of the OH groups (see Scheme 1a) and (ii) pH 11.5 to deprotonate the amine groups and the majority of the phosphonic groups [34]. The amount of AL was adjusted to a nominal fraction of 10 AL/nm^2^ of the NPLs. The solution was heated to 80 °C, and the aqueous suspension of the NPLs was added. The pH did not change significantly because the volumes of the NPL suspensions were relatively small to reach the final reaction concentration of 0.5 mg/mL. The reaction mixture was mixed with a glass stirrer at 80 °C for 3 h and left to cool naturally to room temperature. The product was washed with water five times with intermediate centrifugation at 20,000–50,000 rcf (Superspeed Centrifuge LYNX600, Sorvall, Thermofisher) for 5–10 min, depending on the colloidal stability of the samples. Our final samples were supernatants obtained after the last centrifugation at 2000 rcf for 5–10 min. The coated NPLs were named BHSF-AL.

In addition, we prepared two batches with a nominal fraction of 1.5 AL/nm^2^ corresponding to the theoretical monolayer coverage of the NPLs considering the steric limitation of three phosphonic groups/nm^2^ [35]. The first batch was prepared the same as above and was named BSHF-AL1.5. The second batch was prepared by slow addition of AL to the BSHF NPL suspension (pH 2) preheated to 80 °C. The subsequent steps were the same as for the other samples. This sample was named BSHF-AL1.5-slow.

A parallel series of samples was prepared by washing the as-coated product with NaOH solution (0.1 M, five times) followed by washing with water (three times) with intermediate centrifugation (as above). The aim was to verify the stability of the AL coatings at different pH. 

To verify the processing effect on the core BSHF NPLs, they were treated at pH = 2 and 80 °C for 3 h, similar to the AL coating but without the AL. The samples were named BSHF-80.

Another set of samples was coated with AL (nominal 10 molecules/nm^2^) under hydrothermal conditions at 150 °C for 3 h. All other processing parameters were kept the same as above. The coating pH values were: pH = 5 (one –OH and one –O– per each phosphonic group and NH_3_^+^, similar to pH = 2) and pH = 12 (complete deprotonation of phosphonic and amine groups, similar to pH = 11.5) [34]. The reason for using the less acidic pH = 5 instead of pH = 2 (as above at 80 °C) was to prevent any potential dissolution of the core BSHF NPLs at such extreme conditions. The hydrothermally prepared samples were named HT-BSHF-AL.

### 2.4. Characterization

The as-synthesized and coated BSHF NPLs were analyzed with a transmission electron microscope (TEM, Jeol 2100, Tokyo, Japan) coupled with energy-dispersive X-ray spectroscopy (EDXS; JED 2300 EDS). Dispersed NPLs were drop-deposited on the Cu-supported TEM grid and left to dry.

Electrokinetic measurements (zeta potential) of the NPLs dispersed in deionized water were monitored using a Litesizer 500 (Anton Paar). The pH was adjusted with HCl and NaOH solutions (0.1 or 1 M).

The Fourier transformed infrared (FTIR) spectra of the dried samples were obtained with a universal attenuated total reflectance (ATR) sampling accessory in the range between 4000 and 650 cm^−1^ using a PerkinElmer Spectrum 400 spectrometer (Waltham, MA, USA), and a Bruker Platinum-ATR Alpha spectrometer (Billerica, MA, USA).

Thermogravimetric analyses (TGAs) of AL and dried BSHF NPLs (as-synthesized, treated at 80 °C, and AL coated) were performed with a thermal analyzer and differential scanning calorimet (TGA/DSC 2, Mettler Toledo, Schwerzenbach, Switzerland) coupled with a mass spectrometer (MS, Thermostar 300, Vacuum Pfeifer, Asslar, Germany) for the evolved gas analysis. The samples were heated from 30 to 1100 °C at 20 °C/min in a synthetic air atmosphere with a gas flow of 20 mL/min. The fraction of bonded AL was quantified from the decomposition step of AL at 200–550 °C as previously described in [5]. 

The room temperature magnetic properties of the dried NPLs were measured with a vibrating sample magnetometer (VSM, Lakeshore 7407, Westerville, OH, USA).

## 3. Results

### 3.1. Coatings with APTES

**Figure 2:**
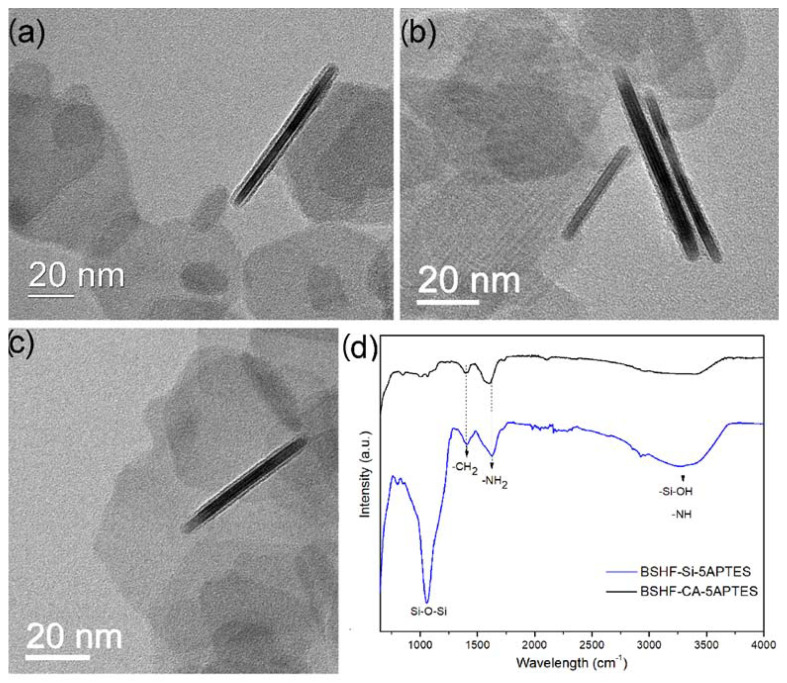
TEM images of (**a**) BSHF-Si; (**b**) BSHF-Si-5APTES; (**c**) BSHF-CA-5APTES NPLs; (**d**) FTIR spectra of samples BSHF-Si-5APTES and BSHF-CA-5APTES.

In the first experiment, we coated BSHF-Si NPLs and BSHF-CA NPLs with 5 molecules/nm^2^ of APTES. TEM images of the coated samples are presented in Figure 2a–c. A 1–2 nm thick amorphous surface layer was observed on all coated samples. The Ms values of the BSHF NPLs were expected to decrease after the coating due to the contribution of the nonmagnetic coating to the total mass of the measured samples. This was true for the BSHF-Si-5APTES sample with Ms = 31 ± 1 Am^2^kg^−1^ in comparison to the core BSHF NPLs with Ms = 35 ± 1 Am^2^kg^−1^. In contrast, Ms = 36 ± 3 Am^2^kg^−1^ for the BSHF-CA-5APTES did not differ from that of the core BHSF NPLs. This can be explained, in part, by the slightly underestimated Ms of the core NPLs due to the adsorbed nitrates (see Section 3.2 for more details) but also by a relatively low mass fraction of the nonmagnetic coating.

The FTIR spectrum of the BSHF-Si-5APTES sample showed characteristic bands at 1161 (Si−O−Si), 1419 (−CH2), and 1626 cm^−1^ (−NH2) and a band at 3000–3500 cm^−1^. This band belonged to –Si−OH or –NH stretching vibrations [36,37]. The BSHF-CA-5APTES sample had almost the same bands, with the exception of the Si−O−Si and –Si−OH bands (Figure 2d). FTIR analysis indicated the amino groups in the coated samples. However, to verify that the amino groups were free on the very surface of the amino-silanized BSHF NPLs, we measured the zeta potential behavior of the aqueous dispersions.

**Figure 3:**
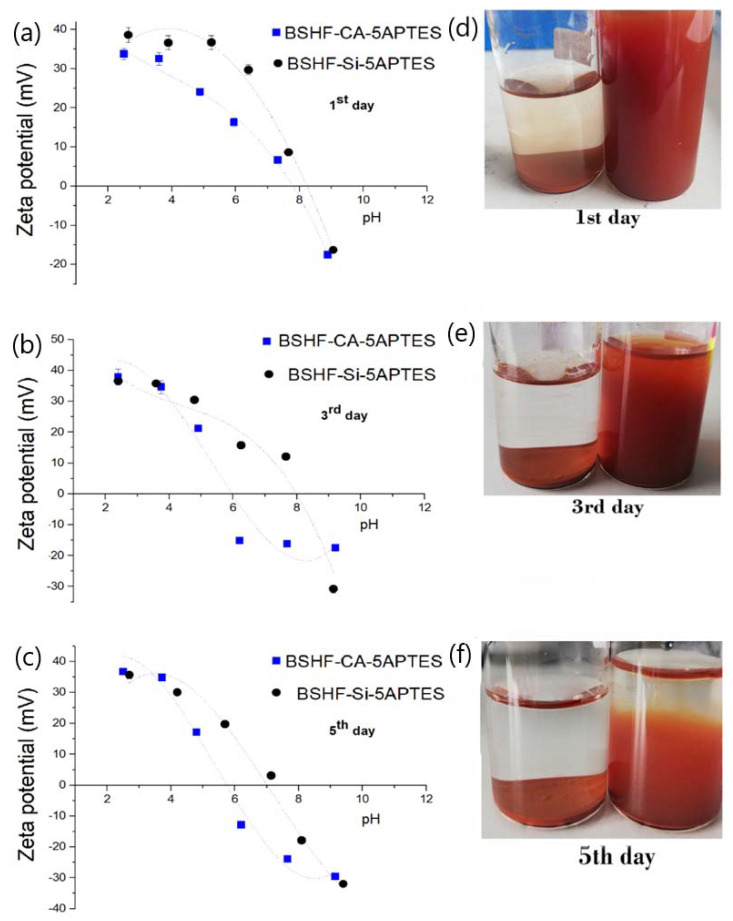
Zeta potential vs. pH of samples BSHF-CA-5APTES and BSHF-SI-5APTES measured: (**a**) immediately after washing (1st day) and on the (**b**) 3rd and (**c**) 5th day after synthesis. The fitting lines in panels (**a**–**c**) are only presented as a guide for the eye. Data were fitted by a 3rd order polynomial fit function. Photos of the measured dispersions were taken on the (**d**) first, (**e**) third, and (**f**) fifth day of synthesis. The vials on the left are sample BSHF-CA-5APTES, and the vials on the right are sample BSHF-Si-5APTES.

The zeta potential, as a function of the pH of the amino-silanized BSHF NPLs, was measured in differently aged dispersions. On the first day of the synthesis, the isoelectric point (i.e., zeta potential = 0 mV) for both samples (i.e., BSHF-CA-5APTES and BSHF-Si 5APTES; Figure 3a) was approximately pH = 8. This was expected since the APTES amino groups should be fully protonated at pH < 9 [22]. After aging the aqueous suspension of the coated NPLs at ambient temperature for several days, a shift in the isoelectric point to lower pH values was observed (Figure 3b,c). The shift was much more pronounced for the BSHF-CA-5APTES sample, indicating a lower stability of the APTES coating, which most likely started to hydrolyze. The inferior coating stability also coincided with the lower colloidal stability of BSHF-CA-5APTES compared to BSHF-SI-5APTES (Figure 3d–f).

**Figure 4:**
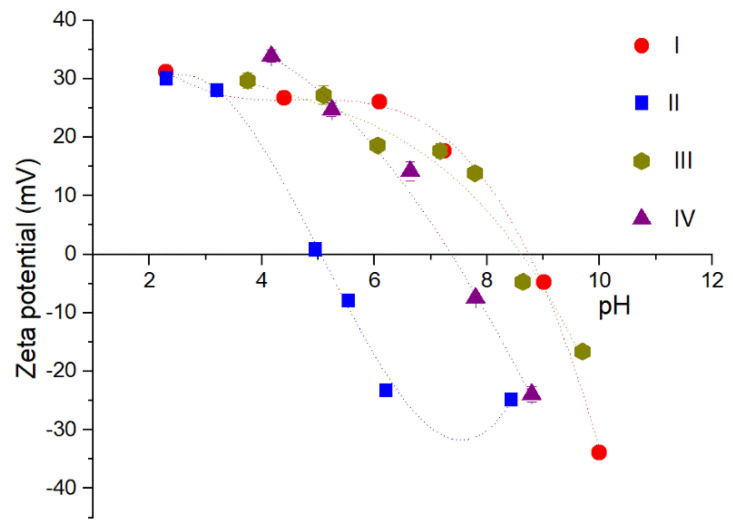
Zeta potential behavior of sample BSHF-Si-30APTES in four batches (I–IV).

In the second experiment, we prepared four batches using the same coating and washing protocol (i.e., water:ethanol mixture; five washing cycles) and a nominal fraction of 30 APTES/nm^2^. We measured the zeta potential of all four dispersions immediately after washing. For the same reaction conditions, we obtained a variety of isoelectric points, from pH = 5 to pH = 8.8 (Figure 4).

APTES forms coatings via a complex mechanism. The silanol groups condense with the hydroxyl groups of the surface metal to form a strong covalent M–O–Si bond. As a side reaction, the reactive silanol species can react with themselves to form oligomers [21]. In addition, the amino groups in the coating compete with the alkoxy moieties for surface sites, and there are few possible variants for intramolecular interactions such as hydrogen bonding and electrostatic interactions [36]. We assumed that using a larger amount of APTES (30 molecules/nm^2^) would ensure the formation of amino coatings; however, we obtained four different isoelectric points, some of which shifted to pH < 8 (Figure 4). Apparently, with the 30 molecules/nm^2^ of APTES, we exceeded its saturation maxima at the BSHF NPLs. Concentrations of APTES higher than a saturation maximum could decrease the isoelectric point, most probably due to the steric crowding that promoted polycondensation and homonucleation of the silane [22].

### 3.2. Coatings with AL

**Figure 5:**
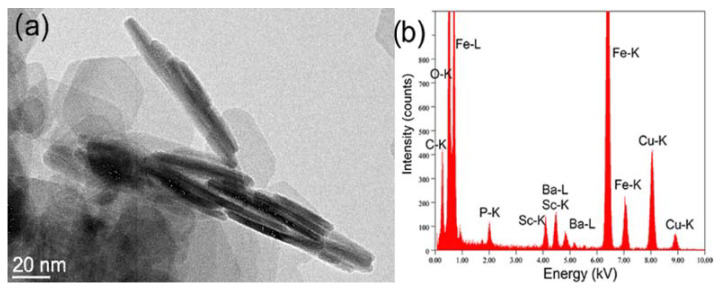
BSHF@AL-1.5-slow NPLs: (**a**) TEM image and (**b**) the corresponding EDXS spectrum. Arrows in panel (**a**) point to an amorphous surface layer on the NPLs perpendicular to the TEM grid. Cu and C in the EDXS spectrum (**b**) originate from the TEM grid, and C can also originate from AL.

The only difference between the AL-coated BSHF NPLs with respect to the as-synthesized NPLs (Figure 1a), observed by TEM, was a thin amorphous surface layer, visible as a blurry surface (Figure 5a). Such a surface layer was observed in all samples, regardless of the coating and washing conditions, including the NPLs heat-treated without AL (i.e., BHSF-80 NPLs). However, there was a distinct difference in the EDXS spectra of differently prepared BSHF@AL (Table 2). While constituent elements of BSHF NPLs (i.e., Ba, Fe, Sc, and O) were detected in all samples, P was detected only in the BSHF NPLs coated at pH = 2 and washed with water (see an example in Figure 5b). The intensity of P was insignificant (i.e., below ±2σ) when the coatings were washed with the NaOH solution. This result suggests the pH-dependent stability of the (surface)Fe–O–P(AL) interaction. Similarly, the intensity of P in the BSHF-AL NPLs prepared in the NaOH solution at pH 11.5 was below the background. Since quantification without a standard, especially for the light elements, such as P, is not reliable with EDXS, the coating composition was assessed from their thermal decomposition products.

We measured TGA coupled with MS for the core and AL-coated NPLs. Mass loss of the core BSH NPLs (~14%) was attributed to water desorption and nitrate decomposition (~9%). The nitrate originated from the aqueous dispersion of core BSHF NPLs in which the pH was adjusted to ensure their colloidal stability with nitric acid. The decomposition of the organic AL part occurred in a broad temperature range of 200–550 °C, after which approximately 50% of the inorganic residue remained up to 1100 °C. This temperature interval was used to determine the mass fraction of AL in the coated NPLs (Table 3). 

**Figure 6:**
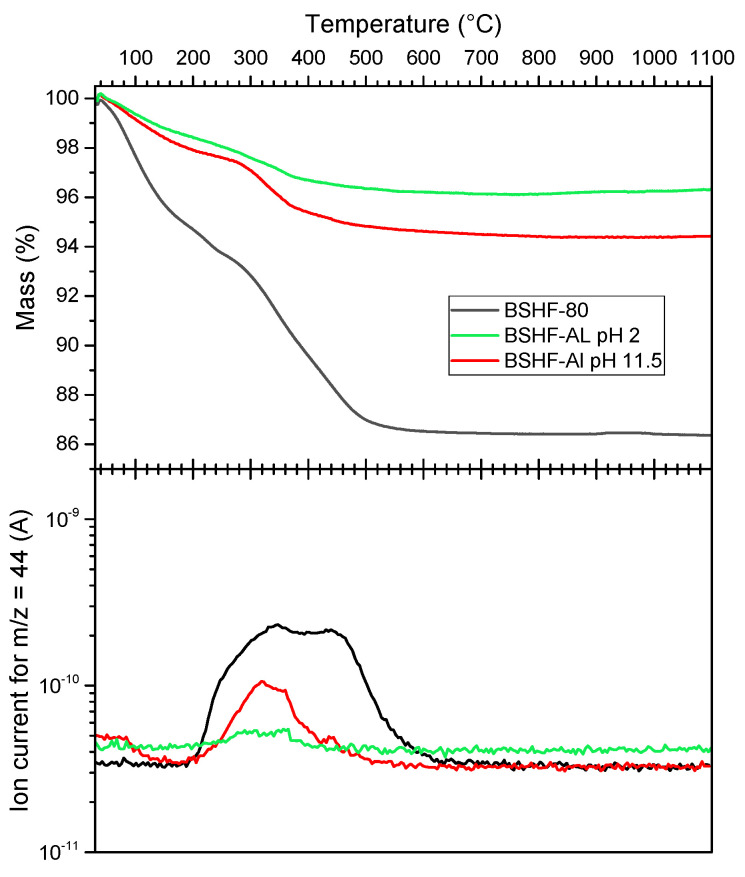
TGA of the BSHF-80 and AL-coated BSHF NPLs (**top**) with the corresponding MS spectra (**bottom**) for m/z = 44 (i.e., CO_2_).

**Figure 7:**
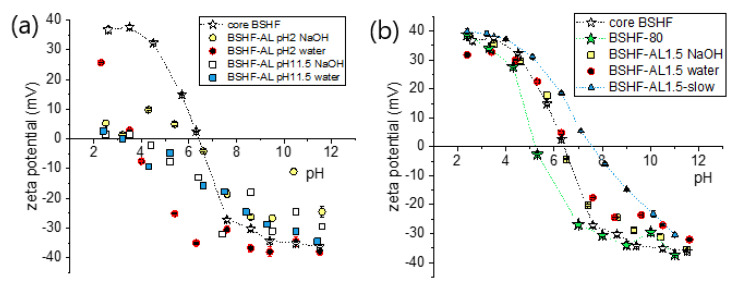
Zeta potential vs pH: (**a**) core BSHF and BSHF-AL NPLs prepared at different pH and washed with NaOH solution or water; (**b**) BSHF-AL-1.5 (pH = 2, washed with water) compared with the core BSHF NPLs that underwent the same treatment. Lines in the panels serve only as a guide for the eye.

Unexpectedly, total mass loss for the measured samples was the largest for the uncoated BSHF-80 NPLs heated at 80 °C and at pH = 2 (Figure 6). According to the MS measurement, most of the mass loss can be attributed to CO_2_ and water. CO_2_ dissolves in water and can adsorb onto the oxide surface in the form of (hydrogen)carbonate. Our result indicates that nitrates were exchanged by the carbonate, which is also in accordance with the lower pH of the isoelectric point for BSHF-80 compared to the as-synthesized core BSHF NPLs (Figure 7a). A very weak CO_2_ peak in the MS spectrum of the BSHF-AL NPLs prepared at pH = 2 indicates that AL exchanged the nitrate from the surface rather than carbonate. Note that AL did not completely decompose up to 1100 °C, which explains the relatively low total mass loss of this sample. The TGA and MS curves of the BSHF-AL, prepared at pH = 11.5, were more similar to those of BSHF-80 NPLs, indicating that the surfaces of the NPLs were predominately carbonated. This is in accordance with EDXS analysis and the low pH of the isoelectric point (Figure 7a). The measured Ms values correspond to the expected Ms values (Table 3) calculated considering the mass fraction of nonmagnetic coating, which proves that our interpretation of the TGA and MS results was correct.

.

The zeta potential behavior was affected by the coating and washing conditions (Figure 7). The isoelectric point of the core BSHF NPLs at pH ~6.5 shifted to a lower pH after coating the NPLs with AL. However, BHSF-AL prepared at pH = 2 and washed with water showed a significantly higher zeta potential (~25 mV) at pH = 3 than all other BSHF-AL samples having a negligible zeta potential at pH < 5. The zeta potential measurements indicate (i) a significantly higher density of AL molecules when they are coated onto BHSF NPLs at pH 2 and washed with water; (ii) that AL does not bind to BSHF NPLs in the NaOH solution at pH ≥ 11.5; consequently, (iii) the AL molecules (partly) detach from the BHSF-AL NPLs when washed with basic NaOH solutions. Unexpectedly, the isoelectric point did not shift to basic pH values for any of the AL-coated samples. Namely, amino-terminated surfaces (e.g., as in Scheme 1b,d) are supposed to shift the isoelectric point to pH > 6.5 (i.e., higher than for the core BSHF NPLs). In contrast, our results indicate that the dominating surface groups were acidic (i.e., phosphonic). 

Based on the above, we propose the following mechanism for the formation of AL coatings on BSHF NPLs. Only one OH group of the two AL phosphonic groups is deprotonated (–O–) at pH = 2 while the amine group is protonated (–NH_3_^+^) [34]. Therefore, AL is in a zwitterion-ion form at pH = 2 (Scheme 1a). As shown previously [38], zwitterions (e.g., amino acids) form associates via electrostatic interaction. Such AL associates: (i) are electrostatically attracted with the deprotonated phosphonic groups to the positively charged surface of BSHF NPLs (Scheme 1c) and/or (ii) chemically bond to the surface (Scheme 1e) via the condensation of protonated phosphonic groups and hydrolyzed surface [27]:

(1) $$R-P-OH\left(aq\right)+crystal–Fe-OH\to R-P-O-Fe-crystal+H_{2}O$$

where *R–P–OH* denotes the AL molecule with the reacting –OH group; *crystal–Fe–OH* denotes the hydroxylated surface Fe^3+^ ions of BSHF NPLs.

The BSHF-AL NPLs prepared at pH = 2 and washed with water were coated with the AL associates (Scheme 1c,e). When the sample was washed with the NaOH solution, the phosphonic and amine groups (almost) completely deprotonated. The negatively charged AL ions were repelled from the negatively charged NPL surface, and they were de-attached and washed away. The neutralization of the suspension by additional washings with pure water enabled the physisorption of the carbonate ions on the free surface. The effect of the washing medium on the surface composition of BSHF-AL prepared at pH = 2 was confirmed with their zeta potential behaviors (Figure 7a) and EDXS analyses (discussed at the beginning of Section 3.2). 

When AL was coated onto the BSHF NPLs at pH 11.5, the phosphonic and amine groups are almost fully deprotonated, and AL was in an anionic form, repelled by the negatively charged surface (i.e., –O–). Due to the absence of protons, the chemisorption (Equation (1)) of AL at the NPL surface was not possible. Consequently, no (or negligible) fraction of the AL ions were adsorbed onto the BSHF NPLs. The unbonded AL was removed during the washing step. When the suspension was neutralized (during the water washing step), carbonate could be adsorbed onto the NPL surface (Figure 6). Consequently, the washing solvent (water or NaOH solution followed by water) had no significant effect on the surface properties of BSHF-AL prepared at pH 11.5 (Figure 7a). 

One way to avoid the association of AL species and inhomogeneous coatings would be to coat the BSHF NPLs with a monolayer of AL, which has been successful for aspartic amino acid [38]. In this case, an amine-terminated surface (Scheme 1b) was expected due to the strong interaction of phosphonic acids with metal-oxide surfaces [25,26,39]. A set of samples was prepared with a nominal fraction of 1.5 AL/nm^2^ (i.e., corresponding to the maximum theoretical density of three phosphonic groups/nm^2^ in a BHF NPL). The BSHF-AL1.5 showed a similar morphology as shown in Figure 5a. Regardless of the very low nominal AL fraction, P was undoubtedly detected by EDXS in the sample washed with water (Figure 5b), while its peak was below the noise when washed with the NaOH solution. However, the zeta potential behavior of the BSHF-AL-1.5 did not differ from that of core BHSF NPLs (Figure 7b), regardless of the washing medium (i.e., water or NaOH solution), as no AL would be adsorbed on the NPLs’ surfaces. This can be explained as follows: nitrate groups adsorbed on the core BSHF NPLs were replaced by the AL to such an extent that the surface density of the acidic groups in total (i.e., phosphonic + nitric) was similar to the density of adsorbed nitrate groups on the core NPLs. Since the amine-terminated surface should increase the pH of the isoelectric point to pH ≥ 8 (Figure 3), we can conclude that the association of AL molecules was not prevented, by lowering their nominal fraction. The above result indicates that electrostatic interaction processes between the AL zwitterions were significantly faster than their interaction with the BSHF NPL surface at 80 °C in water. Indeed, previous work [40] has shown that the condensation of surface metal ions with phosphonic acids is promoted up to 120 °C in the solid state, which is not useful for our case. Namely, any drying of nanoparticles results in strong agglomeration making the surface functionalization of each nanoparticle impossible. Therefore, our experiment was carried out under hydrothermal conditions at 150 °C (see Section 2.3). However, no significant improvement was achieved. 

Finally, to prevent the association of AL, we prepared another batch of BSHF-AL1.5-slow by slowly adding AL to the suspension preheated to the reaction temperature (80 °C) (pH = 2). The isoelectric point of the so-prepared NPL suspension shifted to pH > 7 (Figure 7b), indicating the highest density of the surface amine groups among the samples prepared in this study. In the future, the process can be optimized to increase the surface density of amine groups, e.g., by varying the nominal fraction and the coating temperature.

## 4. Conclusions

We studied the functionalization of ferrimagnetic BSHF NPLs with amino-silane APTES and amino phosphonic acid AL. Both can chemisorb to the oxide surfaces via condensation with the hydroxylated surfaces, resulting in amino-terminated surfaces. The efficiency of the amino-functionalization was assessed from electrokinetic measurements, in particular, by the isoelectric points of differently coated BSHF NPLs, and compared with the core NPLs. The amino-terminated surface was obtained with APTES in a water/ethanol mixture on BSHF NPLs coated with citric acid and silica. However, the coating stability in aqueous dispersions was not optimal for any of the samples. The coatings most likely hydrolyzed slowly and polycondensed homogeneously. The superior stability of the amino-silane coating was achieved on NPLs precoated with silica. The amino-terminated surface was obtained with AL only when it was slowly added at very low concentrations (i.e., 1.5 AL/nm^2^ of the NPLs) at pH = 2 and heated to 80 °C for 3 h. AL did not attach onto the NPLs at high pH when it was (almost) completely deprotonated. The AL coatings obtained at pH = 2 with large AL nominal fractions (i.e., 10 AL/nm^2^) yielded acid-terminated surfaces regardless of the processing conditions. AL is a zwitterion and formed associates that subsequently attached to the NPLs’ surfaces. The associates formed much faster than AL could chemisorb onto the NPLs’ surfaces, and only at suitably low AL concentrations were we able to amino-functionalize the BSHF NPLs. 

## Figures and Tables

**Table 1:** Experimental conditions for coating the BSHF NPLs with APTES.

Sample Name	Nominal Ligand Fraction (APTES/nm^2^)	pH	Washing Solvent
BSHF-CA-5APTES	5	10	Water + EtOH
BSHF-Si-5APTES	5	10	Water + EtOH
BSHF-Si-30APTES	30	10	Water + EtOH

**Table 2:** Experimental conditions for coating the BSHF NPLs with AL.

Sample Name	Nominal Ligand Fraction (AL/nm^2^)	pH	Washing Solvent
BSHF-AL	10	2 or 11.5	water
	10	2 or 11.5	NaOH + water
BSHF-AL1.5	1.5	2	water
	1.5	2	NaOH + water
BSHF-AL1.5-slow	1.5	2	water
HT-BSHF-AL	10 10	5 or 12 5	water NaOH + water
BSHF-80	/	2	water

**Table 3:** Room temperature Ms values of the selected NPLs, all washed with water. The measured Ms values are given with the error estimated from the mass-measurement uncertainty of ±0.1 mg. The Ms values of the core BSHF NPLs were corrected for the mass fraction of adsorbed water and nitrates, as $Ms_{cor}=Ms_{meas}/\left(1-\omega_{water+nitrate}\right)$. The estimated Ms values of the coated NPLs correspond to $Ms_{exp}=Ms_{cor}\times \left(1-\omega_{nonmagnetic}\right)$.

NPLs	*ω_nonmagnetic_* (%)	*ω_ligand_* (%)	*Ms_meas_* (Am^2^/kg)	*Ms_exp_* (Am^2^/kg)
BSHF core	14.35	~9 (nitrate)	28.6 ± 1.0	33 (=*Ms_cor_*)
BSHF-80	13.6	>10 (carbonate)	32 ± 8	29
BSHF-AL, pH 2	8.18	7.21 (AL)	27.9 ± 1.5	30
BSHF-AL, pH 11.5	5.55	~3 (carbonate)	33.3 ± 2.9	31

## Supplementary Materials

The following supporting information can be downloaded at: https://www.mdpi.com/article/10.3390/nano12122123/s1, Figure S1: Selected-area electron diffraction (SAED) of the as-synthesized BSHF NPLs shown in figure 1a. Indices correspond to the magnetoplumbite structure, space group P63/mmc (194).
