# Supplementary material for: New Insights into Amino-Functionalization of Magnetic Nanoplatelets with Silanes and Phosphonates
Source: Nanomaterials (Basel). 2022 Jun 20;12(12):2123. doi: 10.3390/nano12122123 (PMC9229317; doi:10.3390/nano12122123)
Supplement: Supplementary file 1 [file nanomaterials-12-02123-s001.zip › nanomaterials-1774113-supplementary.pdf]

nanomaterials

Supplementary Materials

New Insights into Amino-Functionalization of Magnetic Nanoplatelets with Silanes and Phosphonates

Jelena Papan Djaniš [1,2,*], Griša Grigorij Prinčič [3], Andraž Mavrič [4], Alenka Mertelj [5], Jernej Iskra [3] and Darja Lisjak [1,*]

1 Department for the Synthesis of Materials, Jožef Stefan Institute, 1000 Ljubljana, Slovenia
2 Centre of Excellence for Photoconversion, Vinča Institute of Nuclear Sciences, National Institute of the Republic of Serbia, University of Belgrade, 11351 Belgrade, Serbia
3 Department for Organic Chemistry, Faculty of Chemistry and Chemical Technology, University of Ljubljana, 1000 Ljubljana, Slovenia; grisa.grigorijprincic@fkkt.uni-lj.si (G.G.P.); jernej.iskra@fkkt.uni-lj.si (J.I.)
4 Materials Research Laboratory, University of Nova Gorica, 5000 Nova Gorica, Slovenia; andraz.mavric@ung.si
5 Department of Complex Matter, Jožef Stefan Institute, 1000 Ljubljana, Slovenia; alenka.mertelj@ijs.si
* Correspondence: jelena.papan@ijs.si (J.P.D.); darja.lisjak@ijs.si (D.L.)

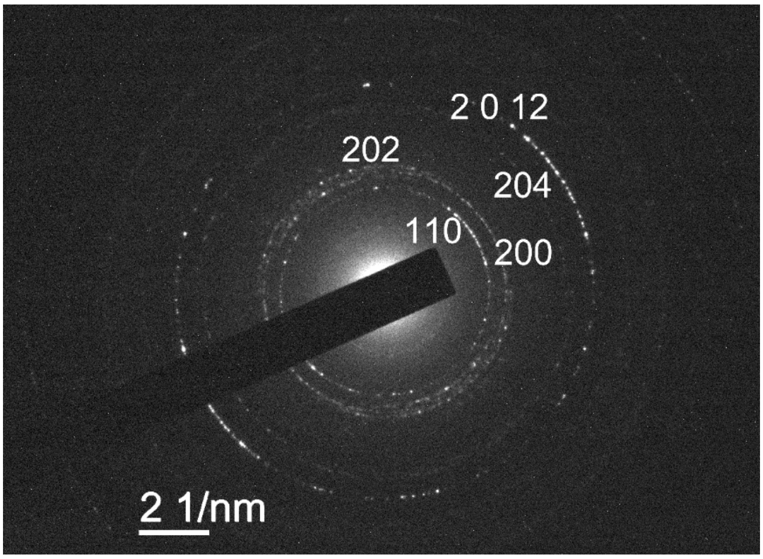

Figure S1. Selected-area electron diffraction (SAED) of the as-synthesized BSHF NPLs shown in Figure 1a. Indices correspond to the magnetoplumbite structure, space group P63/mmc (194).

The surface nominal fraction was calculated from the average size of nanoplatelet (around 50 nm) and thickness (4 nm).

S_{tot} is a total surface of BSHF nanoplatelets;
$S_{nanoplatelet}$ is a surface of single nanoplatelet;
N is a number of BSHF molecules;
m_{tot} is a mass of NPLs core;
$m_{nanoplatelet}$ is a mass of single nanoplatelet,
ρ is a density of BSHF ($5.3 \times 10^3 kgm^{-1}$); r=25 nm is the average radius of a BSHF;
d = 4 nm the average height of BSHF;
V is volume;
N_L is a total number of ligand molecules at surface of NPLs core;
n_L are moles of ligand;
M_L is a molar mass of ligand;
m_L is a mass of added ligand.

$$S_{tot} = N \times S_{nanoplatelet} \tag{S1}$$

$$N = \frac{m_{tot}}{m_{nanoplatelet}} \tag{S2}$$

$$m_{nanoplatelet} = \rho \times V = 5.3 \times 10^3 kgm^{-1} \times \pi r^2 d \tag{S3}$$

$$S_{nanoplatelet} = 2r^2\pi + 2r\pi d \tag{S4}$$

If we add β molecules/ nm^2:

$$N_L = \beta \times S_{tot} \tag{S5}$$

$$n_L = \frac{N_L}{N_{NA}} \tag{S6}$$

$$m_L = n_L \times M_L \tag{S7}$$
